# Failure to Find Altruistic Food Sharing in Rats

**DOI:** 10.3389/fpsyg.2021.696025

**Published:** 2021-06-22

**Authors:** Haoran Wan, Cyrus Kirkman, Greg Jensen, Timothy D. Hackenberg

**Affiliations:** ^1^Department of Psychology, Reed College, Portland, OR, United States; ^2^Department of Neuroscience, Columbia University, New York, NY, United States; ^3^Zuckerman Institute, New York, NY, United States

**Keywords:** social reward, food reward, preference, altruism and prosocial behavior, lever press, rat, social release

## Abstract

Prior research has found that one rat will release a second rat from a restraint in the presence of food, thereby allowing that second rat access to food. Such behavior, clearly beneficial to the second rat and costly to the first, has been interpreted as altruistic. Because clear demonstrations of altruism in rats are rare, such findings deserve a careful look. The present study aimed to replicate this finding, but with more systematic methods to examine whether, and under what conditions, a rat might share food with its cagemate partner. Rats were given repeated choices between high-valued food (sucrose pellets) and 30-s social access to a familiar rat, with the (a) food size (number of food pellets per response), and (b) food motivation (extra-session access to food) varied across conditions. Rats responded consistently for both food and social interaction, but at different levels and with different sensitivity to the food-access manipulations. Food production and consumption was high when food motivation was also high (food restriction) but substantially lower when food motivation was low (unlimited food access). Social release occurred at moderate levels, unaffected by the food-based manipulations. When food was abundant and food motivation low, the rats chose food and social options about equally often, but sharing (food left unconsumed prior to social release) occurred at low levels across sessions and conditions. Even under conditions of low food motivation, sharing occurred on only 1% of the sharing opportunities. The results are therefore inconsistent with claims in the literature that rats are altruistically motivated to share food with other rats.

## Introduction

Pro-social behavior has been defined as behavior that produces benefits for another, sometimes even at a cost to the individual (West et al., 2007; Cronin, 2012; Sosnowski and Brosnan, 2019). One type of pro-social behavior gaining currency in recent years is social release, in which one animal releases another from a trap or restraint. In an experiment by Ben-Ami Bartal et al. (2011), for example, one rat was restrained in a plastic restraint tube that could be opened by a second rat. Such release permitted the restrained animal to leave the tube and spend the remainder of the 60 min session in the presence of the other rat. Most of the rats (17 of 23) learned to open the restraint after an average of about seven sessions. Subsequent studies have verified that rats will, under a variety of conditions, respond in ways that release a rat from a restraint (Ben-Ami Bartal et al., 2014; Silberberg et al., 2014; Sato et al., 2015; Schwartz et al., 2017; Hachiga et al., 2018; Hiura et al., 2018; Blystad et al., 2019; Vanderhooft et al., 2019). The basic effect is reliable, having been replicated across different procedures and laboratories, but its core mechanisms remain a matter of debate.

According to some authors (e.g., Ben-Ami Bartal et al., 2011, 2014; Sato et al., 2015), social release arises from altruistic motives: the free rat senses distress on the part of the restrained rat, and acts altruistically out of empathic concern for its social partner. An alternative explanation is based on social contact: social release is motivated by opportunities for social interaction (Schwartz et al., 2017; Hachiga et al., 2018; Hiura et al., 2018; Vanderhooft et al., 2019). In other words, social release is a type of operant (or instrumental) behavior, established and maintained by contingent social contact as a form of reinforcement. The competing theoretical accounts have been difficult to disentangle experimentally, owing to the fact that under many conditions in the standard procedure, releasing the other rat from a restraint can be viewed in terms consistent with either a social reinforcement view (opportunities for social interaction) or an altruistic view (releasing a distressed rat for its benefit, rather than that of the releaser). Therefore, unless special conditions are arranged to disentangle the two interpretations (cf. Sato et al., 2015; Schwartz et al., 2017; Hachiga et al., 2018), the mere fact of social release is not sufficient to favor one view over the other. (See also Vasconcelos et al., 2012, for a more general critique of the empathy interpretation in a comparative context).

Although most of the work to date on social-release procedures has focused on the main procedure, another result reported by Ben-Ami Bartal et al. (2011) has received far less empirical attention. In some conditions, rats were given a choice between two restrainers, one of which contained high-value food rewards (5 chocolate chips), the other of which contained a restrained rat. These conditions permitted social release, as in the standard procedure, but here, social release was pitted against a known and powerful food reward as a means of assessing the relative value of social release. On average, the rats learned to approach and open the tube containing food more quickly (earlier in the experiment) than the tube containing another rat, though the latencies to access both tubes became low and roughly comparable (<10 s) by the end of the 12-session experiment. And on slightly more than half of the trials, the restrained rat was released before the food was completely consumed, enabling the restrained rat access to the food rewards. This resulted in lower levels of food intake (70% of available food, on average) than in a control condition in which the alternative restraint was empty (in which the free rats ate 96% of available food).

These two patterns of findings led the authors to the following conclusions: (a) the reward value of releasing a restrained rat is comparable to that of high-valued food (owing to the similar latencies to food and social release doors), and (b) social release in some cases comes at the expense of food intake (lower levels of food intake in a social context). In other words, rats not only value social contact equally with food, but engage in altruistic food sharing, sacrificing some high-value food to make it available to a distressed social partner. On its face, these findings appear to lend strong support to accounts appealing to some type of altruism, and simultaneously pose serious theoretical challenges to strict cost-benefit models. If not for altruistic motives, why would a rat sacrifice available food for the good of another? While there is ample evidence of food sharing in rats, it is usually of the reciprocal exchange (tit-for-tat) variety, in which animals alternate roles as donors and receivers (Taborsky et al., 2016). Far less common is the type of unreciprocated food sharing reported by Ben-Ami Bartal et al. (2011), in which a rat leaves highly-valued food for another to consume with no tangible short-term gain for itself. Given both the novelty and theoretical significance of the findings, these food-sharing conditions warrant closer examination.

The main objective of the present study was to replicate and extend the food-sharing conditions from Ben-Ami Bartal et al. (2011), using more robust methods for assessing reward value and food sharing. The methods were patterned after Hiura et al. (2018), in which rats were given repeated choices between high-valued food rewards (sucrose pellets) and social release (10-s social contact). Unlike the findings reported by Ben-Ami Bartal et al., however, Hiura et al. found that rats showed a consistently strong preference for food over social release, even in the face of large increases in food cost (number of responses to produce food), while the costs of social release remained low. Costs were manipulated via a progressive ratio (PR) schedule, in which the number of responses per reward was low at the beginning of the session, but increased with each reward earned. Social release typically only occurred in the latter parts of the session, when food costs were high and after many food rewards had been earned and consumed. The overall session-wide preference for food generally exceeded 90%.

This strong preference for the food over the social release option is at odds with the equal value of social release and food reported by Ben-Ami Bartal et al. (2011). There were several procedural differences between the experiments, however, that may account for the different findings. First, and perhaps most importantly, there were differences in motivation. In the Ben-Ami Bartal et al. experiment, there were no restrictions placed on social or food access outside the session: rats had free access to food and social contact in their homecages. In the Hiura et al. (2018) experiment, on the other hand, food or social access (or both) were generally restricted outside the session. When food was freely available outside the session, the rats still preferred food over social contact, but substantially less so than when it was restricted, suggesting some sensitivity to the motivational context. In the present experiment, we included conditions that both restricted and did not restrict access to food outside the sessions to assess the impact of motivational variables on the relative value of food and social rewards. If the higher levels of food vs. social responding reported by Hiura et al. (2018) are due primarily to motivational variables (restricted post-session access to rewards), then providing free access to those rewards would be expected, through satiation, to reduce the reward value of food, bringing food preferences more in line with social preference, akin to findings of Ben-Ami Bartal et al. (2011).

We also sought a more detailed characterization of altruistic food sharing, the second important claim put forth by Ben-Ami Bartal et al. (2011). In their study, sharing was defined in terms of differences in food consumed when a rat was available for release and when no rat was available (empty tube), differences thought to reflect the amount of food that is consumed (hence, shared) with the other rat. While this provides a tangible measure of some important outcomes of sharing, it has little to say about the functional characteristics of the sharing behavior itself, or the conditions under which it occurs. In the present experiment, we adopted a functional definition of altruistic food sharing, focusing on a coordinated sequence of behavior: (a) food production, followed by (b) social release, given that (c) food was still remaining, permitting (d) the formerly restrained rat access to food. Measuring such episodes of food sharing alongside preference for food and social release will provide important information on how these behavior patterns are related to each other as well as to the experimental conditions.

In addition, to ensure that the choices were well-informed by their outcomes, we gave rats repeated choices each session, rather than the single choice per session used by Ben-Ami Bartal et al. (2011). In single-choice procedures, the duration of social contact depends on when in the session the social release occurs. If the duration of social contact contributes to reward value, as some research suggests (Vanderhooft et al., 2019), then the value of social release in these single-choice procedures may fluctuate across sessions; this may, in turn, affect sharing opportunities. We sought to hold constant the value of social contact, both within and across sessions, and therefore used a consistent (30 s) duration of social contact throughout the present experiment. Against this background of constant value of social reward, we manipulated food quantity (pellets per response) and motivational context (restricted vs. unrestricted food access) on a within-subject basis, including some conditions closely resembling the original procedure used by Ben-Ami Bartal et al. Collectively, the methods permit a rigorous evaluation of Ben-Ami Bartal's two main conclusions bearing on their claim of altruistic food sharing, namely, that (1) reward value of food and social release are equal, and (2) a rat will share food with another rat, even at the expense of food for itself.

## Materials and Methods

### Subjects

Six female Sprague-Dawley rats (*Rattus norvegicus*) were used in this experiment. The rats were experimentally naïve, and were pair-housed in *Ancare*® transparent polycarbonate rodent cages (measuring 26.5 x 48.2 x 20.3 cm) in a temperature-controlled colony room, with a 12 h light/dark cycle. One rat from each pair was designated the unrestrained (focal) rat and the other the restrained rat, where odd-numbered rats 1, 3, and 5 were restrained and even-numbered rats were unrestrained. The rats had continuous homecage access to water whereas the availability of food (*Purina*® Rat Diet 5012) was sometimes restricted (see below). When food was restricted, rats were provided with one hour of food access starting approximately one hr after the conclusion of their experimental session.

### Apparatus

The apparatus consisted of three adjacent chambers, each measuring 31 × 25 × 22 cm with a wire grid floor. The central chamber contained two levers (5 × 1.5 × 1.5 cm) set on either side of a pellet tray, which could receive individual pellets from a gravity-fed dispenser outside the chamber. Additionally, a small light (2 cm diameter) was mounted above each lever. Both the left and right chambers contained a Plexiglas rodent restrainer (25 by 8.75 by 7.5 cm, *Harvard Apparatus*®), each of which was connected to the central chamber by a metal door that was mechanically controlled. Experimenters were able to access each chamber separately via a hatch that, when closed, acted as a ceiling to the chamber. Experimental schedules were controlled and data recorded via a PC computer programmed in the MedState Notation language and MED-PC® software.

### Training

#### Food Reinforcement Training

**Figure 1:**
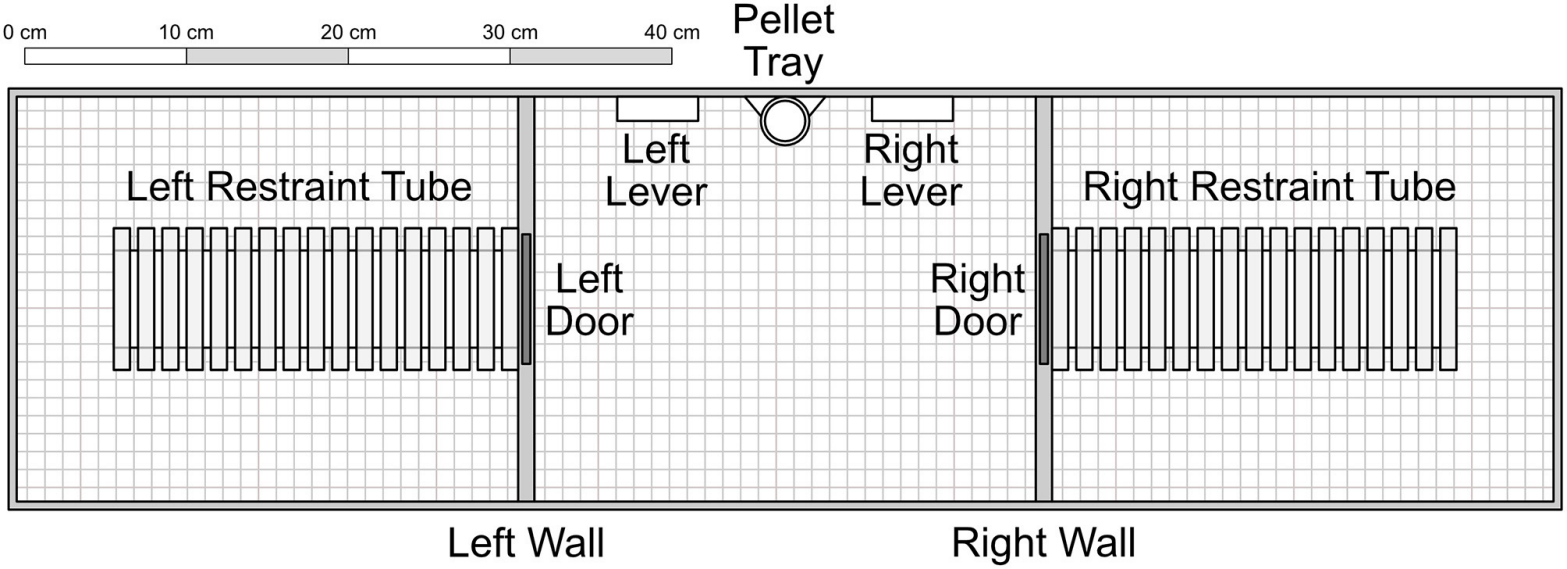
Experimental apparatus, with components presented to scale. The inner left and right walls consisted of opaque plastic. The restraint tubes were cylinders composed of clear Plexiglas. Experimenters accessed each of the three chambers through clear Plexiglas ceiling hatches that acted as a roof when closed. Not shown are two indicator lights set into the wall immediately above each of the levers. Unless otherwise noted, components were composed of aluminum. In Conditions 1–4, pellets were delivered into the pellet tray from a dispenser outside the apparatus. In Conditions 5–7, food was dispensed into the right restraint tube.

Focal rats were trained to press the right lever by reinforcing successive approximations with food, delivered into the pellet tray (see Figure 1), accompanied by a 0.5 s tone. Only the right lever was active in these sessions, denoted by the illumination of the right cue light.

#### Restraint Training

To minimize the delay between lever pressing and social interaction, restrained rats were trained to leave the restraint soon after the door was opened. During these individual sessions, the restraint door was lifted independently of a subject's behavior. Once the rat left the restraint (defined by its entire body, except the tail, having passed through the door) and entered the middle chamber, it was allowed to explore for 30 s before being returned to the restrainer by hand for the next trial. These sessions lasted for 30 min, and continued for 3–5 sessions, until the rats were leaving consistently within a few seconds of when the door was raised. Prior to Condition 6 (see below), restrained rats underwent an additional few sessions of training that introduced the new food delivery location in the right tube restraint. During these training sessions, the restrained rat was placed in the tube on the left and five food pellets were placed in the tube on the right. To initiate each trial, the doors of both the left and right restraint tubes were lifted simultaneously. After 30 s, rats were returned to their restraint, the right tube was refilled with pellets, and a new trial began. Sessions lasted for 30 min, and continued for 2–3 sessions, until the restrained rat consistently entered and ate the available pellets in the right tube. This training was conducted to ensure that the restrained rats would consume food left behind by the focal rat, an important component of our operational definition of sharing.

#### Social Reinforcement Training

After food and restraint training was complete, all rats received social reinforcement training. In these sessions, the restrained rat began each trial in the restraint tube in the left chamber, with the focal rat in the center chamber. Only the left lever was active in these sessions, denoted by the left cue light. A left lever press opened the door to the restraint tube, and produced a 2-s tone. When the restrained rat moved from the restraint into the center chamber, the door was closed, beginning the 30 s social interaction period. This also extinguished the light and deactivated the lever. After 30 s, the restrained rat was returned to the restraint tube for the next trial.

Rat 4 pressed the social lever in the first training session, and therefore required no additional training; left-lever presses only produced social access throughout the experiment. Rats 6 and 8 did not readily press the left lever for social access, and so received two sessions of supplemental training in which left-lever presses produced food. For Rat 6, this brief training was sufficient to establish social responding, and the food contingency on the left lever was withdrawn thereafter. For Rat 8, the food training was also effective in establishing left lever pressing, but responses for social access alone were minimal, compared to the right-lever food responses. To induce more left-lever responses, the contingencies for right-lever responses were made less favorable: 100 responses per food reward (6 sessions) and no rewards/extinction (4 sessions). These changes were successful in producing more frequent social responding, and thereafter the additional contingencies for right-lever responding were discontinued, and the experiment proper began.

### Experimental Procedure

During all experimental sessions, the focal rat in the central chamber made repeated concurrent choices between social release and food, unless otherwise noted. With the exception of Condition 5, choice trials began with both left and right cue lights illuminated and a restrained rat occupied the left restraint. A press on the right (food) lever produced food, with the food quantity, food motivation, and food location varied across conditions (see below). A press on the left (social release) lever opened the left door for a 30 s social interaction period, while also deactivating both levers (and their associated cue lights) for the duration of the interaction period. This prevented the restrained rat from producing consequences arranged for the focal rat. At the end of the social interaction period, the apparatus was reset for the next trial, with the restrained rat returned to the left restraint tube while the focal rat remained in the central chamber. Sessions lasted for a total of 30 min and were conducted five days per week.

A within-subject experimental design was employed, wherein each subject was exposed to experimental conditions in which the main independent variables (food quantity, motivation, food location) were systematically manipulated across blocks of sessions. Table 1 shows the combination of parameters constituting each of the 7 experimental conditions and the sequence in which they were arranged. In Conditions 1–4, food quantity (pellets per press) was increased systematically across conditions: one pellet in Condition 1, two pellets in Condition 2, and four pellets in Conditions 3 and 4. The quantity of food was held constant at 5 pellets per press in Conditions 5–7. Homecage access to food was varied across conditions by restricting access to food to a period of 60 min following the session (Conditions 1–3), or permitting unlimited homecage access (Conditions 4–7).

**Table 1:** Sequence of conditions and number of sessions conducted at each.

**Condition**	**Pellets per response (location of food)**	**Home Cage Chow**	**Food Collection Period**	**# of sessions**		
				**R4**	**R6**	**R8**
1	1 (Tray)	Restricted	N/A	11	11	5
2	2 (Tray)	Restricted	N/A	9	9	4
3	4 (Tray)	Restricted	N/A	13	9	5
4	4 (Tray)	Unrestricted	N/A	5	5	8
5	5 (Right Tube)	Unrestricted	30 s	7	5	7
6	5 (Right Tube)	Unrestricted	Unlimited	6	6	6
7	5 (Right Tube)	Unrestricted	30 s	6	7	5

The location and accessibility of food varied across conditions. In Conditions 1–4, food was dispensed in the pellet tray, in close proximity to the lever, whereas in Conditions 5–7, the food was made available in the restraint tube behind the right door. In these latter conditions, a right lever press opened the right door for a food collection period. In Conditions 5 and 7, this food collection period was 30 s, after which the door closed whether or not all pellets had been consumed. In Condition 6, however, the door remained open until all pellets had been consumed (by either rat). In Condition 5, only the food lever was active; there was no rat in the left restraint tube, the left cue light was left dark, and left lever presses had no mechanical effects. (See Supplementary Videos 1, 2 in the for visual depiction of representative trials in which social or food, respectively, was first produced).

The main dependent variables were (1) proportions of food and social choices made, (2) the number of food and social rewards earned, and (3) the number of pellets either consumed, shared, or left behind. We defined “sharing” as a sequence of behavior consisting of producing food, followed by social release with food remaining, permitting food consumption by the restrained rat. Sharing was possible in all conditions except Condition 5, with no restrained rat present. Food left behind was defined as the difference between the food produced and the total consumed by both rats, and was measured only in Conditions 5 and 7. It was not possible to measure pellets left behind in Condition 6, as trials continued until all food had been consumed.

### Analysis

Performance was modeled using multi-level generalized linear regression, implemented using the Stan programming language (Carpenter et al., 2017). This Bayesian approach yielded a full estimate of the posterior probability density across all relevant parameters, as well as the behaviors those parameters predict. As such, our analysis does not compare behavior to a null hypothesis; instead, we report the patterns of future behavior that are predicted on the basis of the collected evidence, as well as the uncertainty of those predictions given our statistical models. The analytic scripts used to perform these analyses are included in the electronic supplement, as well as an annotated summary of the model assumptions.

## Results

**Figure 2:**
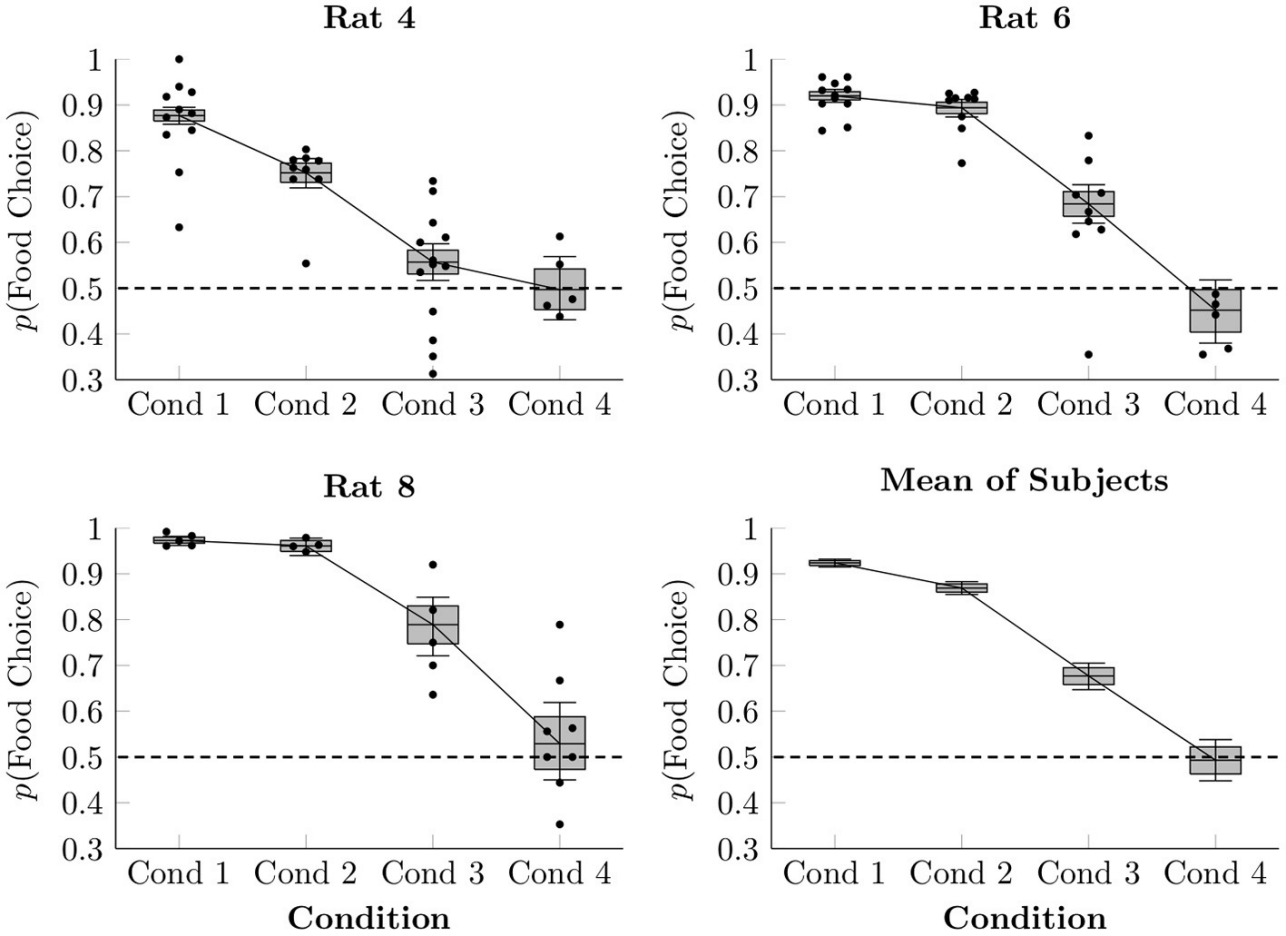
Estimated proportion of food choices made for each of the rats in Conditions 1–4 based on a multi-level logistic regression, as well as observed proportions. The proportion of social choices was complementary to the food choices, such that *p*(*Social Choice*) = 1−*p*(*Food Choice*). Points represent observed means for each session, whereas boxes represent the 80% credible interval and whiskers represent the 95% credible interval of the estimated proportion.

In order to model the proportion of choices made between food and social reinforcement, a multi-level logistic regression was performed, modeling each subject in each condition with an intercept term. The resulting estimated proportion of responses made by each rat to the food lever in each session of Conditions 1–4 is plotted in Figure 2, as are the observed average proportions for each session (points) and the overall estimated mean of the three focal rat subjects (box-and-whisker plots). When access to food in the home cage was restricted (Conditions 1–3), rats consistently favored the food lever over the social lever, doing so most when each lever press yielded only 1 pellet (92.4% mean preference, ± 0.4%), somewhat less when yielding 2 pellets (86.9% mean preference, ± 0.7%), and less still when yielding 4 pellets (67.7% mean preference, ± 1.5%). In Condition 4, with 4 pellets per press and free chow available in the home cage, subjects chose both levers approximately equally over the course of the session (49.3% mean preference, ± 2.3%).

**Figure 3:**
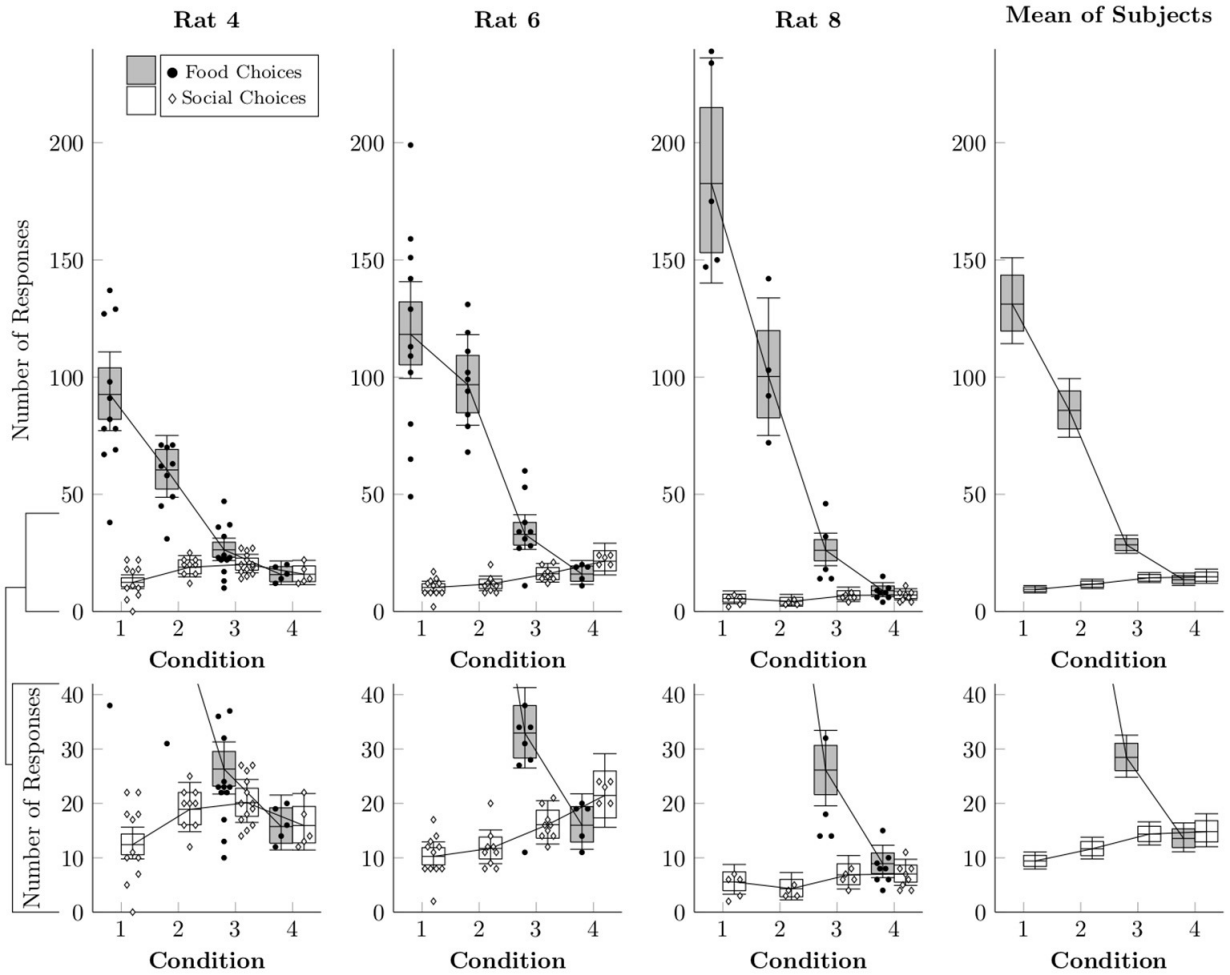
Estimated rates at which food choices (black circles) and social choices (white diamonds) were made for each of the rats in Conditions 1–4 based on a multi-level negative binomial regression, as well as observed event counts. Points represent event counts for each session, whereas boxes represent the 80% credible interval and whiskers represent the 95% credible interval of the estimated rate.

While logistic regression provides an estimate of the relative proportion of food and social choices, it does not reveal the absolute rates of responses to each lever. In order to model the estimated number of food and social choices, a multi-level negative binomial regression was implemented (Gelman et al., 2013). This distribution was chosen because we wanted to allow for the possibility that the distributions were overdispersed. Figure 3 plots the estimated number of times each operandum was chosen per session (box-and-whisker plots), as well as the observed counts of food and social choices for each session (points). Food choices decreased both as a function of number of pellets delivered (Conditions 1–3) and also as a function of free access to chow in the home cage (Condition 4). Social choices, however, did not display any consistent pattern across subjects, happening at similar rates across conditions.

**Figure 4:**
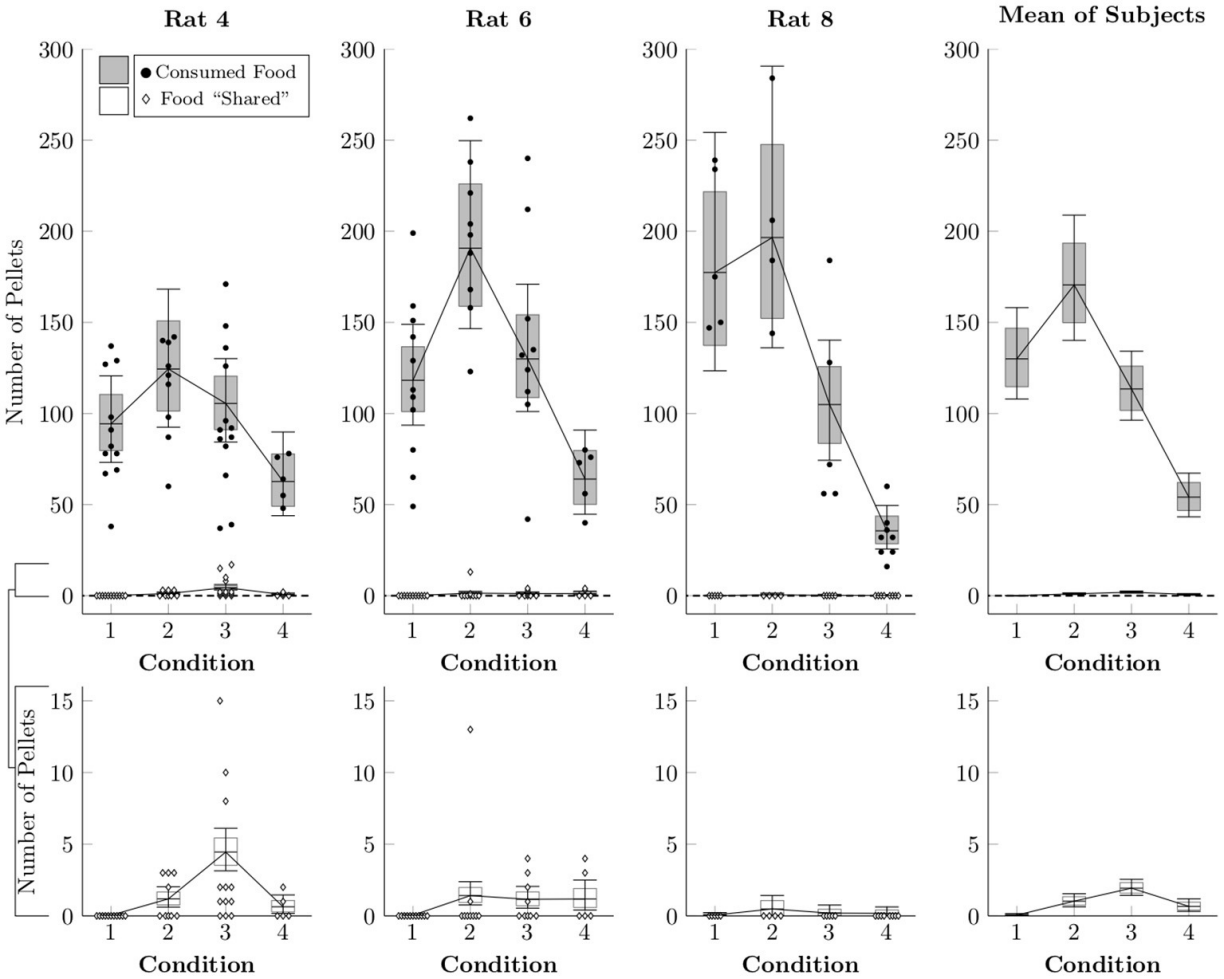
Estimated number of pellets consumed by the focal rat (black circles) and shared with the restrained rat (white diamonds) in Conditions 1–4 based on a multi-level negative binomial regression, as well as observed counts. Points represent number of pellets in each category per session, whereas boxes represent the 80% credible interval and whiskers represent the 95% credible interval of the estimated rate.

Because the rats made frequent food choices and made social choices at consistent rates, there were many opportunities for sharing each session (that is, for producing food pellets, not consuming them, and then releasing the restrained rat). We estimated the rates of both events with another multi-level negative binomial regression. Figure 4 plots the average frequency with which pellets were either consumed (black circles) or left behind (white diamonds) in a given session, as well as the estimated mean rates (box-and-whisker plots). Subjects generally consumed over 100 pellets when access to chow in their home cage was restricted (Conditions 1–3), but consumed around 50 pellets even when they had unrestricted home cage chow (Condition 4). Despite this, the rats effectively never left behind a food pellet in Condition 1 and left behind only one or two pellets in a typical session of Conditions 2–4. Subjects almost never left behind pellets for the restrained rat to collect, even under circumstances in which pellets could be generated easily and during which the focal rat was not experiencing caloric restriction.

This systematic consumption of food may have been stimulus-driven, insofar as subjects pressing the food lever in Conditions 1–4 had the resulting pellets immediately delivered to the pellet tray mere centimeters away from the lever, and any response to the social lever would require the rat to walk past the tray. In Conditions 5–7, however, pellets had to be collected from the right restraint tube, making the pellets both more laborious to collect and less salient as direct consequences of the lever press. Additionally, Conditions 5–7 featured unrestricted home cage chow, so subjects were less motivated by immediate caloric deficit.

**Figure 5:**
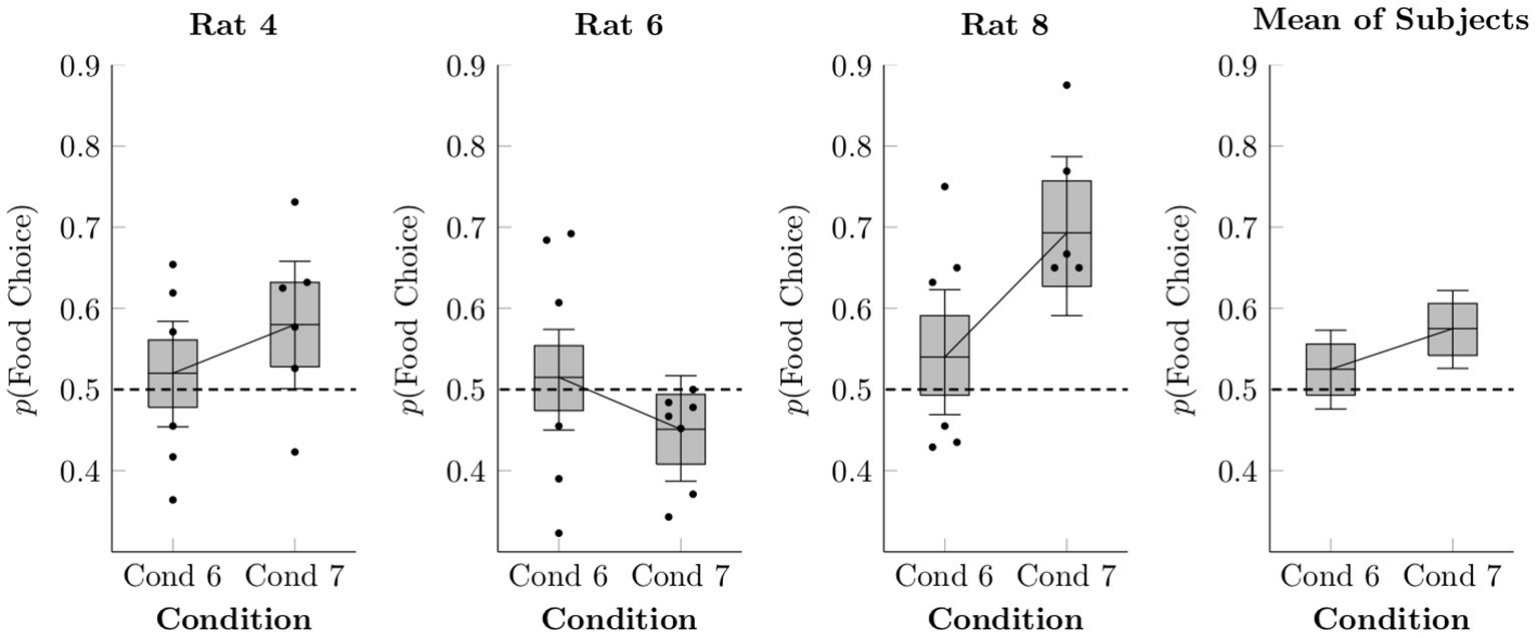
Estimated proportion of food choices made for each of the rats in Conditions 6 and 7 based on a multi-level logistic regression, as well as observed proportions. The proportion of social choices was complementary to the food choices, such that *p*(*Social Choice*) = 1−*p*(*Food Choice*). Points represent observed means for each session, whereas boxes represent the 80% credible interval and whiskers represent the 95% credible interval of the estimated proportion.

Figure 5 shows preference for food responses relative to social responses in Conditions 6 and 7, both as session averages (black points) and as estimated using multi-level logistic regression (box-and-whisker plots). Condition 5 is not present because it lacked a concurrent social schedule. In general, preference was equivocal, close to 50% in both Conditions 6 and 7, at rates similar to those seen in Condition 4 (which also featured free feeding in the home cage). On average, the rats were slightly, but probably not meaningfully, more likely to choose food in Condition 7 than in Condition 6 (mean difference of 5.0% ± 3.5%).

**Figure 6:**
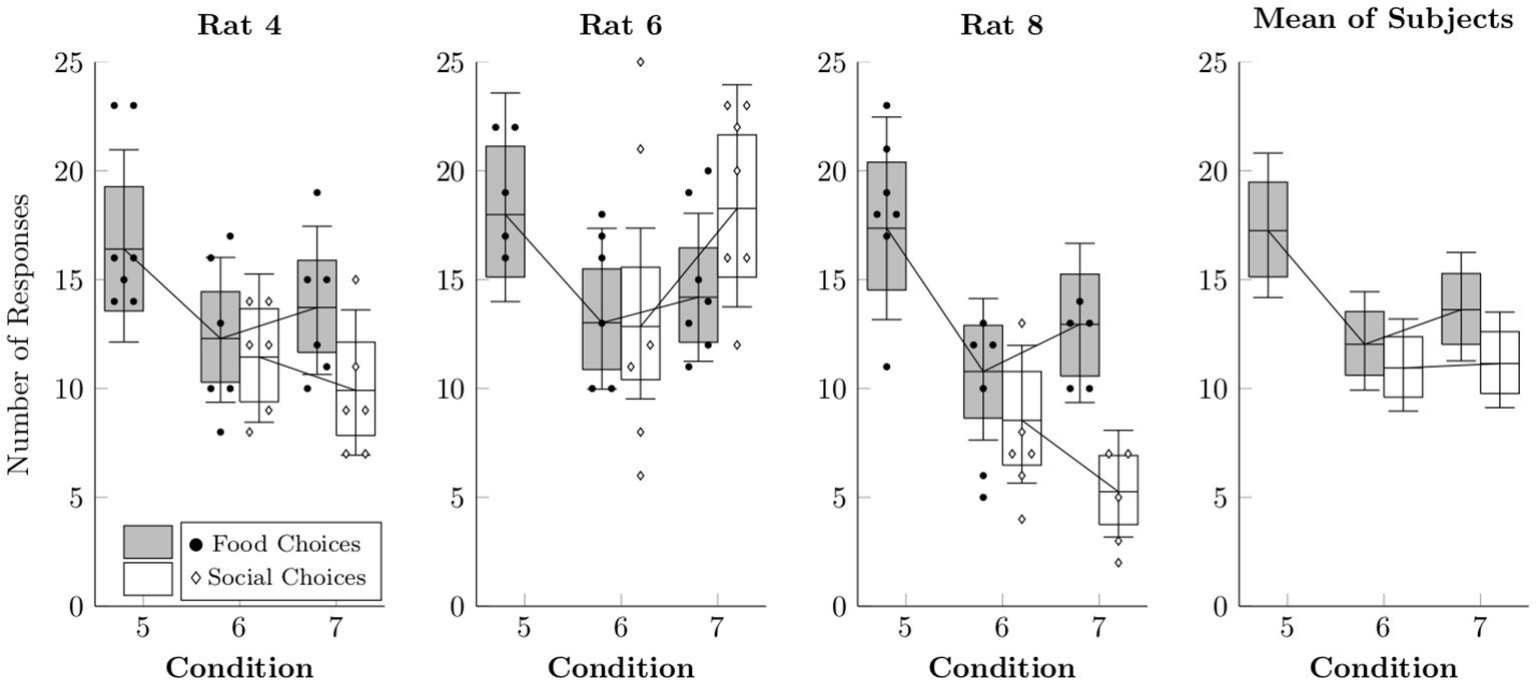
Estimated rates at which food choices (black circles) and social choices (white diamonds) were made for each of the rats in Conditions 5–7 based on a multi-level negative binomial regression, as well as observed event counts. Points represent event counts for each session, whereas boxes represent the 80% credible interval and whiskers represent the 95% credible interval of the estimated rate.

Figure 6 shows the observed counts per session of food choices (black points) and social choices (white diamonds), as well as the rates estimated by multi-level negative binomial regression (box-and-whisker plots). Food choices appeared to happen slightly more often in Condition 5, although this is likely due to the absence of a concurrent choice option. Choices in Conditions 6 and 7 resembled those in Condition 4, suggesting that the alternate food delivery paradigm did not substantially alter response rates to either alternative.

**Figure 7:**
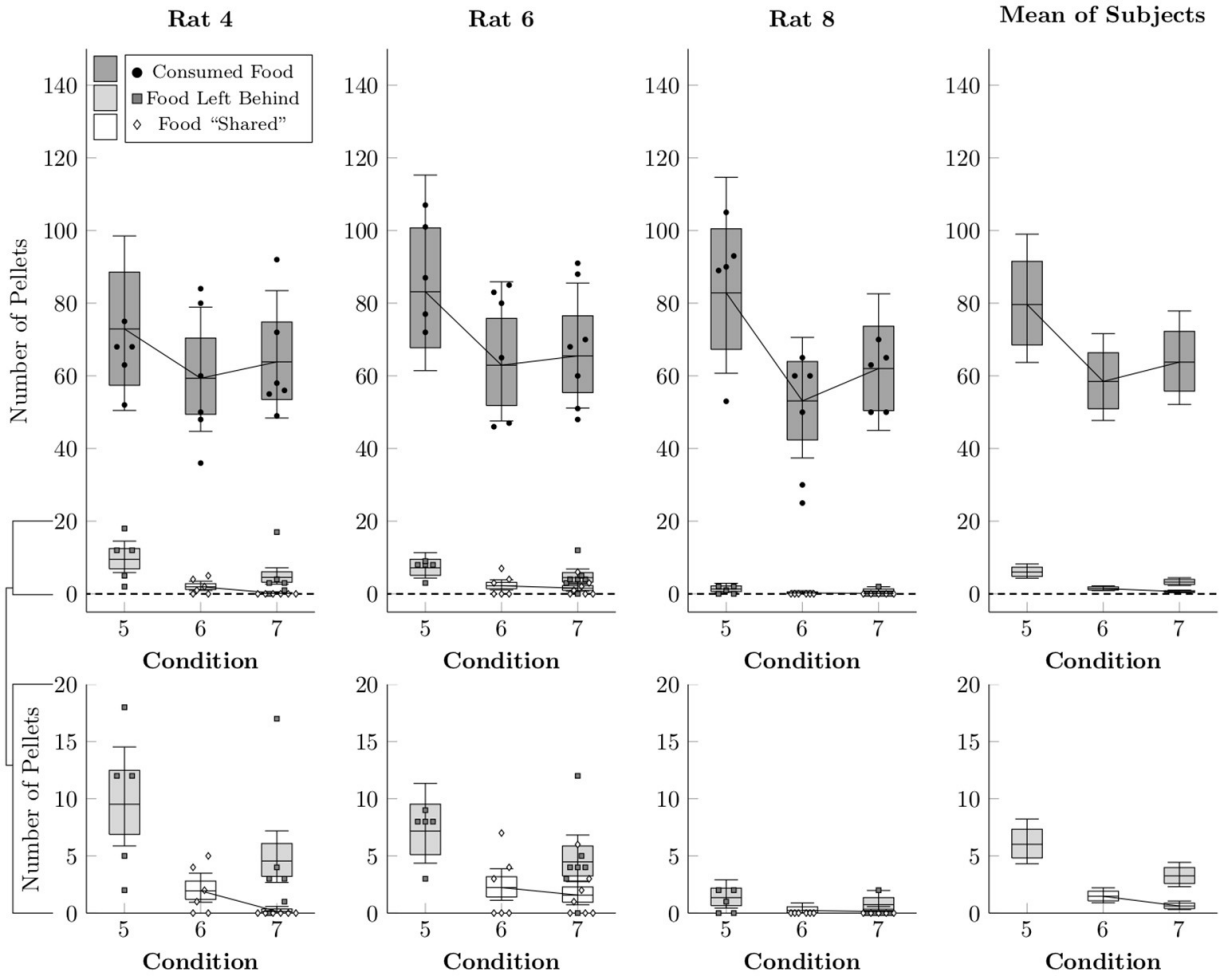
Estimated number of pellets consumed by the focal rat (black circles), shared with the restrained rat (white diamonds), or left unconsumed by either rat (gray squares) in Conditions 5–7, as well as the mean of the subject estimates, based on a multi-level negative binomial regression. Points represent number of pellets in each category per session, whereas boxes represent the 80% credible interval and whiskers represent the 95% credible interval of the estimated rate.

Figure 7 shows the average number of pellets consumed (black points), shared (white diamonds), or left behind (i.e. consumed by neither rat, gray squares) per session in Conditions 5–7, as well as the rates estimated by multi-level negative binomial regression (box-and-whisker plots). As in the earlier conditions, the focal rat tended to consume the vast majority of pellets, but also left quite a few pellets behind due to the time limit on the food collection period in Conditions 5 and 7. In general, more pellets were left behind in Condition 5 (6.0 mean pellets, ± 1.0), than in Condition 7 (3.3 mean pellets, ± 0.5). Sharing, by contrast, happened at low rates in Conditions 6 and 7, comparable to the earlier conditions (1–4). Even given unlimited time to collect pellets, few were shared in Condition 6 (1.5 mean pellets, ± 0.3), and with a 30-s time limit on their collection, even fewer were shared in Condition 7 (0.6 mean pellets, ± 0.2).

## Discussion

The present experiment was designed to replicate and extend some key conditions described by Ben-Ami Bartal et al. (2011), in which rats chose between social release and food. The present research focused on two main findings from that study and their related conclusions: (1) rats chose food and social release with similar latencies, and therefore, food and social release are equally valued; and (2) rats willingly share food with their social partner, even if it comes at a cost to the individual. Taken together, these findings provide key support for the authors' claims of altruistic food sharing. Because occurrences of such unreciprocated food sharing are rare in the published literature (Clutton-Brock, 2009; Taborsky et al., 2016), they warrant further scrutiny.

With respect to the first claim of equal reward value of social release and food, we found that relative value of food and social release varied systematically across conditions. More specifically, when food motivation was low (i.e., the focal rat had unrestricted homecage access to chow in their home cage) and food quantity was high (4–5 pellets per trial), food and social release were chosen about equally often (Conditions 4, 6, and 7), consistent with the (Ben-Ami Bartal et al., 2011) findings. When food motivation was high (restricted access to food outside the session), however, rats clearly preferred food over social release (Conditions 1–3). This finding is consistent with the Hiura et al. (2018) findings, showing strong and reliable preference for food over social release when food is restricted outside the session (see also Blystad et al., 2019). Taken as a whole, the presents results show that relative preference between social and food is not invariant, but rather, is subject to reward and motivational variables (food quantity and overall food access). The relative value of social release and food are always subject to these (and other) variables, and it would therefore be premature to draw broad conclusions about their relative value from sampling only a limited range of conditions. In any case, a motivational view of social and food rewards helps explain discrepant findings from prior research.

The changes in preference across manipulation of food quantity in the first three conditions were driven mainly by changes in the number of food choices per session. This is partly due to economic factors (i.e., decreasing unit price of food) and partly due to satiation. Given the low price (1 response) and the dozens of choice opportunities each session, rats produced and consumed large numbers of sucrose pellets each session when chow in their home cage was restricted (37–284 pellets, mean = 131 across rats). By contrast, when home cage chow was unlimited and food motivation was low, subjects consumed substantially fewer pellets (16–107 pellets, mean = 66 across rats). And when coupled with unlimited food access outside the session in Condition 4, the procedures combined to produce conditions of low food need. Indeed, our rats had such an abundance of food, there was often food left at the end of the food collection periods of Conditions 5–7 (up to 26% of all pellets in Condition 5), even if there was no restrained rat with which to share them. That rats did not consume rewards as highly valued as sucrose pellets suggests a high degree of satiation.

Despite such low levels of food need, there was very little evidence of food sharing – the second and more controversial claim set forth by Ben-Ami Bartal et al. (2011). Behavior that met our operational definition of sharing (i.e., producing food and then releasing the rat while food remained available) was infrequent across all conditions in the experiment, with zero shared pellets being the most common outcome across sessions and the mean being about 1 pellet per session. It did not matter whether food access outside the session was restricted (Conditions 1–3) or not (Conditions 4–7); nor did it matter how many pellets were produced per response (Condition 1–3): rats rarely shared with the other rat any of the abundant supply of food pellets they produced each session. Even in the final two conditions, with procedures that most closely matched the original study (i.e., symmetrically arranged social and food locations, 5 sucrose pellets, and unrestricted access to food and social contact outside the session), sharing was seldom observed (see also Supplementary Video 1). Thus, on the whole, we found no evidence to support the 2011 claim by Ben-Ami Bartal et al. that a rat willingly shares food with another rat.

There is no simple way to reconcile the food sharing reported by Ben-Ami Bartal et al. (2011) with the near complete absence of sharing in the present study. Low levels of food sharing cannot be explained in terms of reduced opportunities for sharing, as the number of social releases (hence, sharing opportunities) remained fairly constant across conditions for individual rats (see Figures 3, 6). This was accomplished by providing repeated exposure to a consistent duration of social contact (30 s) across the experiment. With long sessions and repeated trials, rats had ample opportunities to share the food they had produced; they simply did not do so. The discrepant results also cannot be explained in terms of differing definitions of sharing between experiments. Ben-Ami Bartal et al. (2011) used a less stringent indirect measure of sharing (difference between food consumed with and without a rat available to release) than our behavioral definition of sharing (produce food, then social release with food remaining). This alone cannot be responsible for the different results, however, for even if we adopt the less stringent criterion, our rats showed no differences in food consumption with or without a rat available to release (Figure 7). This is important, as evidence of sharing-related costs are crucial to an altruistic food sharing explanation. Thus, by neither definition did our rats engage in sharing.

We recognize that the present study relies on a small sample size of three rats. Even so, the evidence against food sharing is strong. Across all of the conditions in which sharing was possible, our rats earned an average of 2,171 rewards each (1,662 to 2,772 across rats) of which they shared an average of 47 (0 to 85, across rats), or 1% of the total rewards earned. Because each earned reward provided a sharing opportunity, our rats had vastly greater food sharing opportunities than rats in the Ben-Ami Bartal et al. (2011) experiment. Precise estimates of food sharing opportunities in that experiment are difficult, both because opportunity per reward cannot be derived from the data presented in the paper (% trials with sharing), and because it took the rats several sessions to learn to open the door for either option (before which rewards were not actually available to share). Nonetheless, the theoretical maximum would be 60 food sharing opportunities (five rewards per trial for 12 trials) per rat, roughly 1% of the number food-sharing opportunities in the present study. In addition to vast opportunities for food sharing, the present procedures produced consistent patterns of preferences across animals and over consecutive sessions. Thus, while the small number of rats in the present study limits our ability to generalize to the population of all rats, we have considerable confidence in the results with these particular rats: all were strongly disinclined to share food with their partners across all conditions and thousands of sharing opportunities. Perhaps only some rats engage in altruistic food sharing, differing from their non-sharing conspecifics for some reasons yet to be discovered, and that our sample happened to include only selfish rats who happen to be selfish in very similar ways. This seems unlikely, but it will nevertheless be important to replicate with larger samples of rats in future research.

Another difference between the studies is the food itself. Ben-Ami Bartal and colleagues used a single presentation of 5 chocolate chips, which amounts to approximately 12 calories of food, contained in about 1.62 g. By comparison, each of our sucrose pellets constitutes approximately 0.17 calories, each with a mass of 0.045 g. When subjects had unlimited home cage access to chow, they therefore tended to consume about 11.2 calories of food. Furthermore, rats in Condition 5 (with no opportunity for social access) left about 6 pellets (worth about 1 calorie) behind, despite having no external motivation to do so. This points to subjects with free access to chow leaving high-quality food unconsumed due to satiation, usually doing so shy of 12 calories. If rats with low food motivation are inclined to leave food unconsumed relatively frequently in the absence of conspecifics, it is difficult to argue that losing such food due to sharing can be understood as a “cost.” Ben-Ami Bartal and colleagues give no rationale for their choice of 5 chocolate chips, but based on the patterns of non-social food intake observed in the present study, it seems likely that, had they used 3 chocolate chips, that would have observed almost no sharing, whereas if they had used 7 chocolate chips, they would have observed relatively frequent sharing.

There are other differences between the procedures, and the only way to know for certain which factors are responsible for the discrepant results would be to begin with a direct replication, an exact reproduction of the original procedures, and thereafter change one variable at a time. We chose instead to conduct a systematic replication (Sidman, 1960), in which some, but not all, of the original procedures are reproduced. Systematic replications are useful in assessing the generality of a finding, and this fit with our broader objectives of providing a more thorough characterization of preference and sharing. We sought not only to replicate but to extend, to assess the generality of the findings by exploring behavior across a range of conditions, including but not limited to, those of the original study. In particular, the lack of adequate control conditions leaves the original study open to multiple interpretations. Sampling independent variables under varying conditions puts replication efforts into a broader context, changing the focus from binary questions with yes-no answers (e.g., Do rats value social release over food? Do rats share food with another rat?) toward conditional questions (e.g., Under what conditions is social release favored over food, and vice versa? Under what conditions does sharing occur?). Viewed in this way, Ben-Ami Bartal and collaborators are not so much incorrect as they are interpreting incomplete evidence; their results are part of more general relationships between preference and sharing and the variables of which they are a function.

Exploring such functional relationships across a parametric range can also shed light on theoretical disputes. For example, when examined at only a single point on a function, social release can be interpreted either in terms of social reward (response-contingent access to social interaction) or in terms of empathy (acting out of concern for the other rat): both accounts make the same prediction that door opening will occur. The accounts begin to differ, however, as behavior is examined while other experimental parameters change. For example, in procedures similar to those used here, Vanderhooft et al. (2019) first trained social release in rats, then systematically increased the price of social release (number of responses to produce it) across sessions, generating demand functions. Overall, the functions (27 in all) were well-described by the Hursh and Silberberg (2008) essential value model, a model that has proven useful in quantifying the value of numerous other rewards, including food, water, and drugs (Hursh and Roma, 2016). In other words, rates of social release behavior were predictable, with a high degree of quantitative precision, on the basis of these social reward functions. It is less clear, however, what, if anything, an empathy account would have to say about these data: it makes no obvious predictions about how empathy is affected by price – or other variables known to affect reward value (e.g., magnitude, delay, or probability), about which social reward makes clear and testable predictions. And if predictions could be derived from an empathy account (e.g., by assuming that empathy mirrors social reward functions), they would be indistinguishable from the more parsimonious social reward account, and would therefore add little to the explanation. This is not to deny the importance of empathy as a topic worthy of scientific study; it is, rather, to demand more stringent tests of it, especially in domains in which simpler explanations already exist.

## Data Availability Statement

The original contributions presented in the study are included in the article/Supplementary Material, further inquiries can be directed to the corresponding author.

## Ethics Statement

The animal study was reviewed and approved by Reed College Institutional Care and Use Committee.

## Author Contributions

The study was conceptualized and designed by HW, CK, and TH. Data were collected by HW and CK. Data were analyzed by HW, CK, and GJ. The paper was written by HW, CK, GJ, and TH. All authors contributed to the article and approved the submitted version.

## Conflict of Interest

The authors declare that the research was conducted in the absence of any commercial or financial relationships that could be construed as a potential conflict of interest.

## Supplementary Material

Supplementary Video 1 Video from a representative trial from Condition 6, with concurrent choice between food and social release. The focal rat first selected the food option, and consumed all 5 food pellets from the tube, ending the trial. Had some or all of the food pellets been left, the trial would have continued, thereby permitting sharing. (the camera angle is opposite the front of the apparatus, reversing the Left/Right orientation shown in Figure 1).

Supplementary Video 2 Video from the trial subsequent to that shown in Video 1, with concurrent choice between food and social release. The focal rat first selected social release, which permitted 30 s of social interaction, ending the trial. (the camera angle is opposite the front of the apparatus, reversing the Left/Right orientation shown in Figure 1).
