# Supplementary material for: Failure to Find Altruistic Food Sharing in Rats
Source: Front Psychol. 2021 Jun 22;12:696025. doi: 10.3389/fpsyg.2021.696025 (PMC8259949; doi:10.3389/fpsyg.2021.696025)
Supplement: Supplementary file 1 [file Data_Sheet_1.zip › Data and analysis code/Model specifications.pdf]

Supplementary material
Model Specifications

Each of the following annotated model summaries describes the statistical assumptions made by our models, using the notation described by Gelman et al. (2013).

Model for Proportion of Food Choices (Figures 2 & 5)

$F_i \sim \text{Binomial}(N_i, p_{s,c})$

The number of food choices F_i made out of N_i opportunities is governed by a binomial distribution with a probability $p_{s,c}$, which is a separate value for each subject in each condition.

$p_{s,c} = \text{Logistic}(\beta_{s,c})$

The parameters describing subjects' performance are encoded as unbounded scalar parameters that are converted to probabilities by the logistic function.

$\boldsymbol{\beta} \sim \text{MVNormal}(\boldsymbol{\mu}, \boldsymbol{\Sigma})$

$\boldsymbol{\beta}$ is a Subjects-by-Conditions matrix whose values are drawn from a multivariate normal distribution. A subject's mean across all conditions is given by the corresponding element in the vector of means $\boldsymbol{\mu}$.

$\mu_s \sim \text{Normal}(0,1.5)$

The prior for each subject's mean μ_s is normally distributed.

$\boldsymbol{\Sigma} \sim \text{diag}(\boldsymbol{\tau}) \cdot \text{LkjCorr}(2) \cdot \text{diag}(\boldsymbol{\tau})$

The covariance matrix $\boldsymbol{\Sigma}$ is obtained by construction using a vector of magnitudes $\boldsymbol{\tau}$ and the LKJ Correlation distribution (Lewandowski et al. 2009).

$\tau_s \sim \text{Exponential}(1.5)$

The prior for each subject's covariance magnitude τ_s is exponentially distributed.

Model for Proportion of Total Responses Made (Figures 3 & 6)

$R_i \sim \text{NegBinomial2}(\mu_{s,c,t}, \phi)$

The total number of responses R_i is governed by a negative binomial distribution using the alternate parameterization whose mean is μ and whose variance is $\left(\mu + \frac{\mu^2}{\phi}\right)$. Each subject has two parameters in each condition: One governing food responses and one governing social responses (if available).

$\mu_{s,c,t} = \text{Log}(\beta_{s,c,t})$

The parameters describing subjects' performance are encoded as unbounded scalar parameters that are made strictly positive by a log transformation.

$\boldsymbol{\beta} \sim \text{MVNormal}(\boldsymbol{\gamma}, \boldsymbol{\Sigma})$

$\boldsymbol{\beta}$ is a Subjects-by-(Conditions×2) matrix whose values are drawn from a multivariate normal distribution, such that each condition yields two parameters per subject (one for food responses and one for social responses). A subject's mean across all conditions is given by the corresponding element in the vector of means $\boldsymbol{\gamma}$.

$\gamma_{s,t} \sim \mathrm{Normal}(1,1.5)$

The prior for each subject's mean for each response type $\gamma_{s,t}$ is normally distributed.

$\boldsymbol{\Sigma} \sim \mathrm{diag}(\boldsymbol{\tau}) \cdot \mathrm{LkjCorr}(2) \cdot \mathrm{diag}(\boldsymbol{\tau})$

The covariance matrix $\boldsymbol{\Sigma}$ is obtained by construction using a vector of magnitudes $\boldsymbol{\tau}$ and the LKJ Correlation distribution (Lewandowski et al. 2009).

$\tau_{s,t} \sim \mathrm{Exponential}(1.5)$

The prior for each subject's covariance magnitude for each response type $\tau_{s,t}$ is exponentially distributed.

$\phi \sim \mathrm{Exponential}(1)$

The negative binomial overdispersion term has an exponential prior and is shared across subjects.

Model for Proportion of Total Pellet Counts (Figures 4 & 7)

The model for counting the total pellets either consumed, shared, or left behind followed identical logic to the negative binomial regression described above, with two exceptions. First, it provided three parameters per subject per condition, rather than two, in order to account for the three different observed counts. Second, the prior for γ_s was set to $\mathrm{Normal}(0,1.5)$ in anticipation of the lower counters for all three cases.
